# Serological responses to a soluble recombinant chimeric *Plasmodium vivax* circumsporozoite protein in VK210 and VK247 population

**DOI:** 10.1186/1475-2875-12-323

**Published:** 2013-09-14

**Authors:** Yang Cheng, Daisuke Ito, Jetsumon Sattabongkot, Chae Seung Lim, Deok-Hoon Kong, Kwon-Soo Ha, Bo Wang, Takafumi Tsuboi, Eun-Taek Han

**Affiliations:** 1 Department of Medical Environmental Biology and Tropical Medicine, Kangwon National University School of Medicine, Chuncheon, Gangwon-do 200-701, Republic of Korea; 2 Proteo-Science Center, and Venture Business Laboratory, Ehime University, Matsuyama, Ehime 790-8577, Japan; 3 Mahidol Vivax Research Unit, Faculty of Tropical Medicine, Mahidol University, Bangkok 10400, Thailand; 4 Department of Laboratory Medicine, College of Medicine, Korea University, Seoul, 152-703 Republic of Korea; 5 Department of Molecular and Cellular Biochemistry, Kangwon National University School of Medicine, Chuncheon, Gangwon do 200-701, Republic of Korea

## Abstract

**Background:**

Circumsporozoite protein (CSP) is essential for sporozoite formation and sporozoite invasion into human hepatocyte. Previously, a recombinant *P. vivax* CSP based on chimeric repeats (rPvCSP-c) representing two major alleles VK210 and VK247 within central region has been designed. Naturally acquired humoral immune responses study show that antigenicity of rPvCSP-c was much higher than that of native strain. However, the serologic reactivity of rPvCSP-c was still unclear in detail.

**Methods:**

In present study, recognition of rPvCSP-c in vivax malaria typed VK210 and VK247 alleles was assessed. VK210 typed and VK247 typed sera from adult residents reacted specifically with rPvCSP-c using protein array and immunoblot assay. Additionally, anti-rPvCSP-c serum recognized the fixed VK210 and VK247 sporozoites by immunofluorescence assay. Furthermore, statistic analysis was performed for correlational detection.

**Results:**

The rPvCSP-c reacted with both VK210 typed and VK247 typed *P. vivax* infected patient sera and anti-rPvCSP-c immune serum also reacted with VK210 and VK247 sporozoite parasites of *P. vivax* specifically. There was a positive correlation between increased antibody level, age of patients and also associated with *pvcsp* repeat number, although the level of responses did vary considerably in their reactivity to the rPvCSP-c from negative to very high level within each age group.

**Conclusions:**

These data confirmed the serologic reactivity of the novel rPvCSP-c in exposed both VK210 and VK247 populations. These results strongly suggested that this recombinant CSP was biologically active and potently immunogenic across major strains and raised the prospect that this protein could be used as serologic marker.

## Background

All species of *Plasmodium* shows that circumsporozoite protein (CSP) contain a central repeat region, whose amino acid sequence is species-specific, and its functional role of the repeat sequence is recognition of species-specific host receptors [1,2]. The repeats contain B-cell immunodominant epitopes [3] and antibodies specific to this region can protect against malaria by blocking sporozoite invasion of host hepatocytes [1]. In particular, the central repeat region of *Plasmodium falciparum* CSP, which contains an immunodominant B cell epitope, represented the target of the first two vaccine trials [4,5]. The repeat region of *Plasmodium berghei* CSP, alone, is unable to mediate sporozoite infectivity in either the mosquito or the mammalian host [6].

PvCSP contains three major alleles, VK210, VK247, and *P. vivax*-like including one of three types of nonapeptide repeat units, GDRA(A/D)GQPA, ANGA(G/D)(N/D)QPG and APGANQ(E/G)GGAA, respectively [7-11]. Both VK210 and VK247 of which distributed widely all of the world [12-16], although the antigenicity of VK210 and VK247 suggested recognition of VK210 variant was higher than recognition of VK247 [13,17,18]. *Plasmodium vivax*-like CSP was detected in samples from Brazil, Madagascar, and Indonesia [10,11,19]. However, a number of globally collected blood samples were fail to detect *P. vivax*-like CSP, but were positive for VK210, VK247, or both [20].

In optimization of serological marker, sensitivity of antigen appeared to be essential. Apical membrane antigen 1 (AMA1), merozoite surface protein 1 C-terminal (MSP-1_19_) and CSP have been used in *Plasmodium* species as serological markers [21-24]. Highly polymorphic antigens were not an ideal choice because the prevalent morphs in the community of interest could differ from those of the test antigen, which leaded to underestimation of seroprevalence rates [25]. Different prevalence of antibodies to CSP suggested that seroprevalence might be genetically determined [26,27]. These potential limitations should be taken into account for designing serological marker.

In a previous study, naturally acquired humoral IgG immune response study showed that the antigenicity of rPvCSP was higher than that of native protein [28]. However, as global serologic marker, the serologic reactivity of rPvCSP was still unclear in detail. In present study, human sera from patients of both VK210 and VK247 parasites infection reacted to the conserved chimeric rPvCSP (rPvCSP-c), and demonstrated correlation with patient age and parasitaemia in responsiveness. Interestingly, although there was no typical difference of responsiveness among different repeats, here was a tendency of negative correlation between repeat number and serum responsiveness.

## Methods

### Patient samples

One hundred six VK210 typed serum samples were obtained from patients who were confirmed positive for vivax malaria via microscopy at local health centers and clinics in Gyeonggi and Gangwon Provinces near Demilitarized Zone (DMZ) of the Republic of Korea (ROK); Korean DMZ is an approximate 250 km long buffer zone between North and South Korea which runs along the 38th parallel north. Being close to North Korea, the first reemerging case was diagnosed here, and almost high-, medium- and low-risk malarial areas were located at least 10 km from the DMZ [29]. Eight VK247 typed serum samples were obtained from symptomatic, smear-positive patients from Mae Sod, Thailand, where is a town in western Thailand that shares a border with Burma. It is notable as trade hub and for its substantial population of Burmese migrants and refugees. Furthermore, eighty serum samples were taken from healthy residents in Gyeonggi and Gangwon Provinces, but far from DMZ, who confirmed negative for vivax malaria by microscopy, and these were used as controls. To confirm rPvCSP-c specific reactivity, twenty *P. falciparum* patient serum samples from Uganda were used as control. This study was approved by the Institutional Review Board at Kangwon National University Hospital.

### Construction and expression of rPvCSP-c

The synthetic PvCSP gene was constructed as a chimera based on the amino acid sequence of PvCSP-VK210 (Belem strain) and PvCSP-VK247 (PNG strain). The synthetic CSP gene designed in the present study consisted four parts; i) conserved N-terminal seventy one amino acids of VK210, ii) four VK210 (Belem strain) repeat regions, iii) three VK247 (PNG strain) repeat regions, and iv) conserved C-terminal seventy amino acids of VK210. The classical repeat in VK210, GQPAGDRAD, was represented three times as well as KQPGDRAD once started from conserved N-terminal. In addition, a triple copy of the classical VK247 repeat, GANGAGNQP, was included in the construct. This was followed by the conserved C-terminal region and ended at amino acids TDVC, before the additional six histidines tag. Production of rPvCSP-c using a wheat germ cell-free (WGCF) expression system, which using previously described bilayer translation reaction methods [30,31]. The rPvCSP-c was purified using a Ni-nitrilotriacetic acid agarose column under non-denature condition (Qiagen, Valencia, CA, USA) as described elsewhere [32].

### SDS-PAGE and Western blot analysis

The rPvCSP-c was separated by SDS-PAGE under reducing conditions. The separated proteins were transferred to 0.45 μm PVDF membranes (Millipore, Billerica, MA, USA) in a semi-dry transfer buffer (50 mM Tris, 190 mM glycine, 3.5 mM SDS, 20% methanol) at a constant 400 mA for 40 min using a semi-dry blotting system (ATTO Corp., Tokyo, Japan). After blocking with 5% skim milk in phosphate buffed saline containing 0.2% Tween 20 (PBS/T), mouse anti-penta His antibody (Qiagen), *P. vivax*, *P. falciparum* or sera from healthy individuals (1:200) diluted into PBS/T, which were collected from ROK, Thailand and Uganda, and secondary IRDye® goat anti-mouse (1:10000, PBS/T) or IRDye® goat anti-human (1:20000, PBS/T) (LI-COR® Bioscience, USA) were used to detect His-tagged recombinant proteins. Data was scanned by Odyssey infrared imaging system (LI-COR Biosciences, Nebraska, USA) and analysed by Odyssey software (LI-COR Inc. Lincoln, Nebraska, USA).

### Immunization of mice with rPvCSP-c

Six- to eight-week-old female BALB/c mice (DBL Co., Seoul, ROK) were injected intraperitoneally with ~20 μg of rPvCSP-c and PBS as negative control with Freund’s complete adjuvant (Sigma-Aldrich, St. Louis, MO, USA). Three mice were used per group. Booster injections were given after three and six weeks using the same amount of antigen with Freund’s incomplete adjuvant (Sigma-Aldrich). Mouse blood samples were taken two weeks after the final boost.

### Serum screening using protein arrays

Sera from 114 vivax malaria patients, 20 falciparum malaria patients and 80 healthy individuals were tested against the rPvCSP-c using protein arrays as previous report [33]. Briefly, one microlitre of rPvCSP-c (6 ng/μl) was spotted to each well of an amino-functionalized slide and incubated for 2 h at 37°C. The arrays were firstly blocked with 5% BSA in PBS/T for 1 h at 37°C. Then they were probed with human serum (1:10) that was pre-absorbed against wheat germ lysate (1:100) to block anti-wheat germ antibodies. The arrays were incubated with serum in PBS/T for 1 h at 37°C and antibodies were visualized with 10 ng/μl Alexa Fluor 546 goat anti-human IgG (Invitrogen, Carlsbad, CA, USA) in PBS/T and scanned in a fluorescence scanner (ScanArray Express, PerkinElmer, Boston, MA, USA) [34]. Fluorescence intensities of array spots were quantified by the fixed circle method using ScanArray Express software (version 4.0, PerkinElmer). The positive cut-off value was calculated as the mean fluorescence intensity (MFI) value of the negative controls plus 2 SD. For the correlation between the antibody reactivity against rPvCSP-c (MFI) and parasitaemia of vivax sample, forty nine known parasitaemia samples were used and analysed. Mean intensity > 10,000 was seen as high intensity, and > 0.2 seen as high parasitaemia.

### Indirect immunofluorescence assay (IFA)

Sporozoites were obtained from the salivary glands of *Anopheles dirus* mosquitoes approximately 17 to 21 days after the blood meal and typed for the strain of *P. vivax* (*P. vivax* 210 and 247 types) [35]. The phenotype of sporozoite was defined by PCR then using RFLP analysis specific 210 and 247 as previous description [36]. Sporozoites were coated onto multi-well slides, air dried, and fixed with ice-cool acetone. Slides were blocked with non-fat milk diluted to 5% in PBS at 37°C for 30 min. Anti-rPvCSP-c serum, diluted in PBS as 1: 200, was added to the wells, and the slides were incubated in a humidified chamber for 1 h at 37°C. Meanwhile, only PBS immunized serum, diluted in PBS as 1:200, also was used to develop IFA as negative control. The slides were washed with PBS and Alexa Fluor-488-conjugated goat anti-mouse antibody (Invitrogen), and nuclear strain with DAPI (4’, 6-diamidino-2-phenylindole) (Invitrogen) was added for 30 min at 37°C. The slides were mounted in ProLong Gold antifade reagent (Invitrogen) and visualized under oil immersion in a confocal scanning laser microscope (LSM5 PASCAL; Carl Zeiss MicroImaging, Thornwood, NY) using a Plan-Apochromat 63×/1.4 oil differential interference contrast (DIC) objective lens. Images were captured with LSM5 PASCAL software and prepared for publication with Adobe Photoshop (Adobe Systems, San Jose, CA, USA).

### Statistical analyses

Simple scatter-regression was used for making standard curve by SigmaPlot (Systat Software Inc., San Jose, CA, USA). IgG antibody response to rPvCSP-c and the correlation coefficient between antibody titers and parasitaemia percentage were analysed using GraphPad Prism (GraphPad Software, San Diego, CA, USA). Spearman’s rank correlation was used to evaluate correlations between the variables. Student *t*-test was used to compare the differences between the means of each group for statistical significance. Statistical differences of *p* < 0.05 were considered significant.

## Results

### Expression of rPvCSP-c

**Figure 1:**
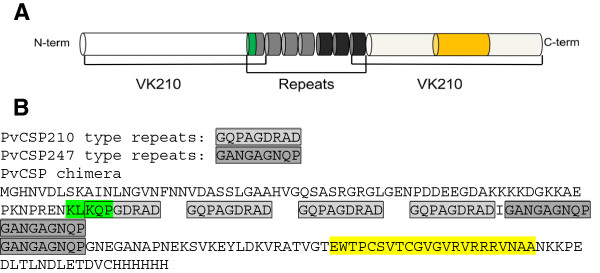
**Protein structure (not drawn to scale) and amino acid sequence of the recombinant PvCSP molecule.** The repeat region is flanked by N-terminal and C-terminal conserved regions based on the VK210 sequence. The central region represents four VK210 repeats (grey) and three VK247 repeats (black), region I and region II indicated with green and yellow respectively **(A)**. Totally, 205 amino acids consist of this PvCSP chimera including four VK210 repeats (gray), three VK247 repeats (dark), region I (green) and region II (yellow) **(B)**.

Highly conserved repeat sequence in central region of *pvcsp* gene exists in other *P. vivax* parasites. VK210 and VK247 types are two distinct forms of PvCSP, which are different in their central repeat region, distributing all of world. Although *P. vivax*-like CSP variants is one of major alleles in Brazil, *P. vivax*-like CSP variants is much less popular than VK210 and VK247 in worldwide endemic areas. Therefore, in order to produce a recombinant *P. vivax* serologic marker that encompasses the predominant antigenic forms of the CSP observed in the different regions, A chimeric CSP, including N- and C-terminal, and truncated repeat region of VK210, and truncated repeat region of VK247 was constructed (Figure 1A). This rPvCSP-c is comprised of four repeat regions from VK210 and three repeat regions from VK247 (Figure 1B).

**Figure 2:**
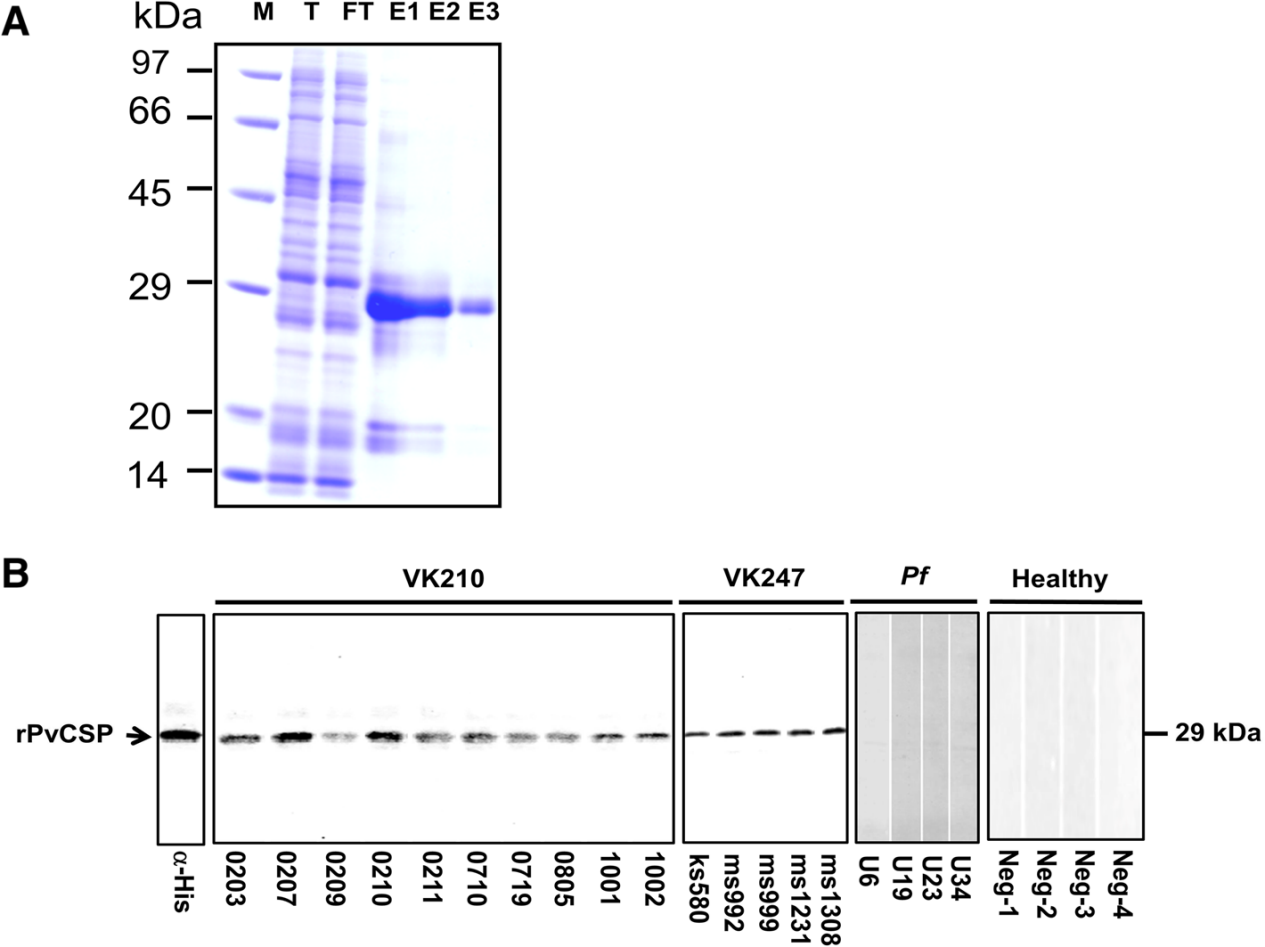
**SDS-PAGE of recombinant *P. vivax *CSP (rPvCSP) and its immunoblot reacted with human sera.** The size of the rPvCSP was 30 kDa and was estimated by comparison with known molecular weight standards **(A)**. Purified rPvCSP was separated by SDS-PAGE under reducing condition and electroblotted onto PVDF membrane **(B)**. The blot was blocked and reacted with Penta-His antibody, VK210 typed patient serum samples from ROK adults (numbered samples) or VK247 typed patient serum from Thailand (ks580, ms992, ms999, ms1231 and ms1308); *P. falciparum* infected patient serum from Uganda (U6, U19, U23 and U34) and sera from vivax naïve individuals in ROK (Neg-1, Neg-2, Neg-3 and Neg-4) as negative control. T: total translation mix; FT: flow through; E: elusion; M: molecular mass marker.

Chimeric recombinant PvCSP was expressed as a soluble His-tag fusion protein of 29 kDa. This product was an abundant soluble protein expressed by WGCF expression system, and was successfully purified by using of Ni-sepharose column under non-denature condition (GE Healthcare, Camarillo, CA, USA) (Figure 2A).

### Reactivity of *P. vivax* infected serum samples to rPvCSP-c

Sera from individuals exposed to *P. vivax* were used to determine whether the rPvCSP-c had epitopes reactive with antibodies induced from a naturally occurring infection. For the initial analysis, serum was collected at random from a small number of adult patient samples of VK210 and VK247 types. Twenty-three individual serum samples were used for these assays, including 10 sera from vivax patients in ROK (VK210 type), 5 sera from vivax patients in Thailand (VK247 type), four sera from falciparum patients in Uganda and with four negative serum samples without exposure to malaria (Figure 2B). Additionally, anti-His antibody was developed to confirm rPvCSP-c purity and reactivity by Western blot analysis. Each serum sample was incubated separately against purified rPvCSP-c that was size fractionated by SDS-PAGE and blotted onto PVDF membrane. *Plasmodium vivax*-exposed individuals had antibodies that reacted with rPvCSP-c, but sera from the *P. faciparum*-exposed or unexposed donors did not suggesting that antibody of VK210 and VK247 typed serum samples specific to rPvCSP-c.

### Recognition of rPvCSP by VK210 and VK247 allelic sera

**Figure 3:**
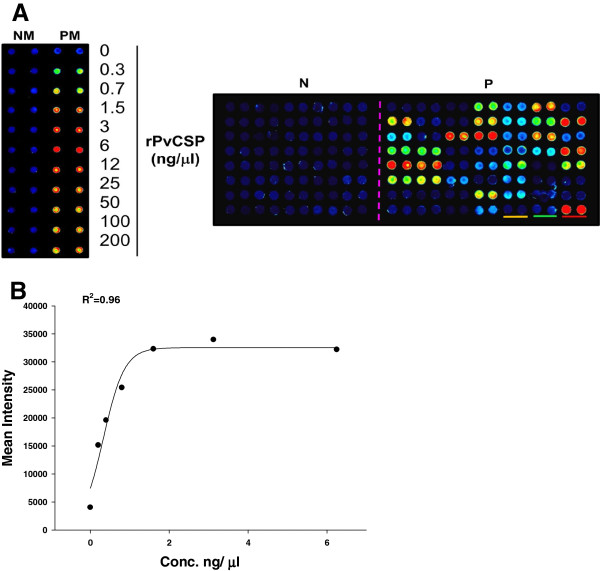
**Development of a protein array platform for profiling antibody responses to *P. vivax *infection. (A)** Left panel. Different concentrations of rPvCSP were probed with pooled human serum (left duplicate, negative serum; right duplicate, positive serum). Right panel. rPvCSP (6 ng/μl) reacted with individual serum samples in duplicates. Left phase of slide: vivax negative serum samples; Right phase of two slides: vivax positive serum samples. Red bar, pooled positive sera reacted with rPvCSP; Green bar, pooled vivax negative sera reacted with rPvCSP; Yellow bar, 10 OD wheat germ extract reacted with rPvCSP. NM: negative *P. vivax* patient sera mixture; PM: positive *P. vivax* patient sera mixture; N: individual negative serum; P: individual positive serum. **(B)** Correlation between spot intensities and the concentration of rPvCSP.

**Figure 4:**
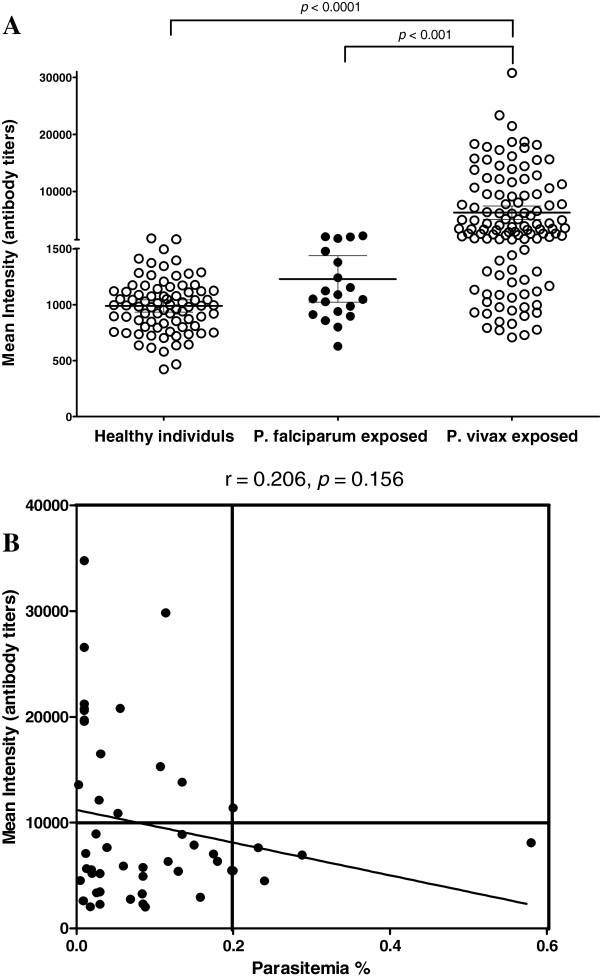
**IgG antibody responses to recombinant PvCSP using protein microarrays. (A)** Probing rCSP with sera from vivax or falciparum malaria patients and from healthy individuals. Each circle indicates the data from individual serum analysed. The bar indicates mean value plus standard deviations. The *p* value was calculated by Student’s *t-*test. **(B)** Forty nine known parasitaemia samples were analysed the correlation between the antibody reactivity against rPvCSP and parasitaemia of vivax sample. Correlations were evaluated using Spearman’s correlation test. Mean intensity > 10000 seen as high intensity, and > 0.2 seen as high parasitaemia.

In order to analyse the prevalence of natural infection-induced immune response, IgG reactivity in 106 VK210 and 8 VK247 allelic serum samples from Thailand and Republic of Korea, respectively. Optimal concentration (6 ng/μl) of protein coating for amino-functionalized slide was determined using pooled sera from healthy samples and vivax malaria patients was shown in the left panel of Figure 3A. A concentration-dependent analysis method showed a correlation coefficient (*R*^2^ = 0.96) between fluorescence intensities and protein concentrations (Figure 3B). The efficiency of protein arrays for antibody profiling was evaluated using purified rPvCSP-c (Figure 3A, right panel). A range of titers were observed, in which *P. vivax* infected patient samples were significantly higher than that of falciparum patient serum samples (*p* < 0.001) and vivax negative serum samples (*p* < 0.0001), although there was still an apparent cross-reactivity with *P. falciparum* which might be because of conserved regions of CSP in *Plasmodium* species (Figure 4A) [37].

Protein arrays detected *P. vivax* in 83 of 114 sera samples from the microscopically positive samples, thus, sensitivity was 72.8%, among these protein arrays detected positive samples, 75 of 106 VK210 sera samples (70.8%) and 8 of 8 VK247 sera samples (100.0%) (Table 1). From the eighty sera samples from healthy subjects, three false positives were obtained by protein arrays, thus, specificity was 96.2%. There was significant difference between the fluorescent intensity of vivax patient serum samples and healthy serum samples response to rPvCSP-c (*p* < 0.0001) (Table 1). To better determine the serological responses to rPvCSP-c, whether 106 VK201 patient ages correlate with response were examined. When the subjects were divided into age groups (< 20, 20 – < 40, ≥ 40 years or < 25, 25 – < 50, ≥ 50 years) significant differences were seen in serologic responses to rPvCSP-c (Table 2). There was a wide range in patients’ responsiveness to the rPvCSP-c antigen that had a positive correlation with age (*p* < 0.05). The level of antibody seen by the average and median responses increased dramatically with age as expected. These results indicated that rPvCSP was highly antigenic and was recognized by sporozoite-induced antibodies. This could infer that rPvCSP might develop high prevalence of recognition in serological screening.

**Table 1:** Prevalence (% positive), 95% confidence intervals, and MFI values of IgG responses to rPvCSP-c in sera typed VK210 and VK247 vivax malaria patients and healthy individuals

**Type group**	**No. of patients samples (*n*)**			**95% CI (%)**^**b**^	**MFI**
	**Positive**	**Negative**	**Total (%)**^**a**^		
VK210	75	31	106 (70.8)	61.5–78.6	5883
VK247	8	8	8 (100)	67.6.1–100	11198
Total	83	31	114 (72.8)	64.0–80.1	6256
**Type group**	**No. of healthy samples (*n*)**			**95% CI (%)**^**d**^	**MFI**
	**Positive**	**Negative**	**Total (%)**^**c**^		
Total	3	77	80 (3.8)	89.6–98.7	989

^a^Sensitivity: % of Positive in patient samples.

^b^Confidence intervals.

^c^Specificity: % of Negative in healthy samples.

^d^Differences in the total IgG prevalence for each antigen between vivax patients and healthy individuals were calculated with Student’s-t test. *P* < 0.05 considered as statistically significant.

*MFI* mean fluorescence intensity.

**Table 2:** Seroprevalence to the rPvCSP-c in residents of ROK (VK210), observed by two different age groupings

**Age group (years)**	**No. of samples**		**Fluorescence intensity**
	***n***	**% positive**	**Mean ± SE**^**a**^
Group 1			
< 20	17	35.3	2,483 ± 542
20 – < 40	68	76.5	6,296 ± 807^b^
≥ 40	21	81.0	7,299 ± 1322^c^
Group 2			
< 25	32	46.9	4,158 ± 898
25 – < 50	58	81.0	7,182 ± 897^d^
≥ 50	16	81.3	6,494 ± 1466
Total	106	70.8	5,883 ± 602

^a^Group mean antibody responses are given as log transformed antibody units. Values ≥ 1476 were considered positive. Student *t*-test was developed to different groups.

^b^*p* < 0.05 and ^c^*p* < 0.001 compared with < 20 in group 1.

^d^*p* < 0.05 compared with < 25 in group 2.

*P*< 0.05 was considered significant. *SE* standard error.

To investigate whether patient parasitaemia associated with antibody titers, we would predict a direct relationship between the vivax patient parasitaemia and the response of vivax serum sample against rPvCSP-c. However, for none of each serum sample titer (intensity) as well as parasitaemia tested was a tight correlation observed (r = 0.206, *p* = 0.156, Figure 4B), although a general grouping of results based on the patient parasitaemia correlate to serum intensity was noted. Nevertheless, in no sample was there high titer processing in the high parasitaemia (Figure 4B, top right quadrant), may indicate that, when the antibodies against PvCSP did inhibit parasite growth, it was contributing in some degree to the overall inhibition of parasite growth. In contrast, high antibody titer of patient samples only involved into low parasitaemia although low antibody titer also involved into low parasitaemia.

### Analysis of the correlation between immune response in Korean VK210 and its repeat number

In present study, due to these VK247 typed samples contain only 1 repeat region and Korean indigenous types were closer to VK210 than VK247 [38], therefore 20 Korean VK210 typed samples from 106 positive samples were sequenced and analysed and all of their intensity were higher than cut-off value, as a result, eight samples including 20 repeats, seven samples including 18 repeats and five samples including 24 repeats within their CSP central region (Table 3). Although there was no significant difference (*p* > 0.05) among three repeat groups, here is negative correlation between repeat number and patient immune response.

**Table 3:** Seroprevalence to the rPvCSP-c in residents of ROK (VK210), observed by three different repeat number groupings

**No. of repeat in each group**	**No. of samples**		**Fluorescence intensity**	
	***n***	**% positive**	**Mean ± SE**^**a**^	***p *value**^**b**^
18	7	100	19,930 ± 4,509	-
20	8	100	18,550 ± 4,173	0.82
24	5	100	14,510 ± 3,327	0.39
Total	20	100		

^a^Group mean antibody responses are given as log transformed antibody units.

^b^Analyse *p* value by comparing with 18 repeats group. Values ≥ 1476 were considered positive. Student *t*-test was developed to different groups, comparing 20 repeats and 24 repeats with 18 repeats respectively. *P* < 0.05 was considered significant. *SE* standard error.

### Assessment of biological activity of antibodies induced by rPvCSP-c

**Figure 5:**
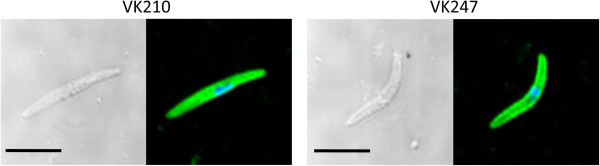
**Indirect immunofluorescence microscopy with anti-rPvCSP sera on the *P. vivax *sporozoites.** Mouse antiserum to rPvCSP was incubated with smears of VK210 sporozoites (VK210) and of VK247 sporozoites (VK247). In both VK210 and VK247 sporozoites, the anti-rPvCSP serum reactivity was shown in green and nuclear staining with DAPI was shown in blue (right panels). Bright field DIC images were presented in the left panels. Black bar indicates 5 μm.

To assess whether antibodies generated against this chimeric molecule were able to recognize native surface protein of sporozoite parasites, immunofluorescence assays were performed with VK210 and VK247 sporozoites of *P. vivax*. Sera from mice immunized with rPvCSP-c had strong immunofluorescence reactivity with both type of sporozoites (Figure 5) and preimmune serum had no reactivity.

## Discussion

*Plasmodium vivax* has two distinct forms of the CSP designated VK210 and VK247 types, which differ in the sequence of the central repeat region [7]. rPvCSP-c has a truncated repeat region that incorporates the reported variant repeat sequence combinations of VK210 and VK247 repeat sequences. As putative serologic marker, rPvCSP-c encompassed the predominant antigenic forms of the CSP present in the different geographical regions was confirmed. *Plasmodium vivax*-like CSP, as another major allele, also was detected in samples from Brazil [10,11,19]. However some further study failed to detect this allele in global blood samples [20]. In addition, one examination from around 1700 samples in Thailand showed that VK210 prevalence was much higher than that of VK247, but no *P. vivax*-like variant allele detection [39]. Intriguingly, a similar strategy as described in the original report of *P. vivax*-like CSP was used to identify such allele, they were unable to detect any variant type other than VK210 or VK247 type in *P. vivax* samples from Thailand [14]. Nevertheless, all repeat units present in the CSP were not necessary to induce effective immunity [40,41]. Therefore, in present study, a molecule without *P. vivax*-like CSP region, but diverse number of repeats of PvCSP was designed for producing effective antigenic reactivity. As described before, the antigenicity of VK210 variant was higher that of VK247 variant [13,17,18]. All together, rPvCSP-c encompassed the N-terminal and C-terminal of VK210 flanking a chimeric repeat region representing VK210 and VK247, the two major alleles of PvCSP as shown in Figure 1. This CSP-based serologic marker might be able to target all of the field isolates with the help of adequate T- and B-cell epitopes. However, the rPvCSP-c may have detection limit for *P. vivax*-like variant in Brazil, where the *P. vivax*-like variant frequency was higher than VK247 type frequency [10,11] and that *P. vivax* distribution can be different in other areas of America as Colombia, Mexico and Asia.

In present study, thirty-one false negative and three false positive recognitions were detected in vivax-infected patients and healthy samples, respectively (Table 1). Although the reason of this phenomenon was still unclear, it was not rare that patients exposed to *P. vivax* blood infection do not harvest anti-CSP antibodies especially if the infection was caused by relapse episode may cause the false recognitions. Recently, lower immunogenicity of VK247 peptide had been analysed [42], and antibodies to VK210 were more frequent than those to VK247 [13]. Due to the prevalence of VK247 was not as high as VK210 type in endemic countries except limited endemic countries from previous report [43], only few VK247 serum samples tested and the source of the samples was limited to one geographic site in present study, it was necessary to test more VK247 serum samples from different areas for further evaluating rPvCSP-c serologic marker potential in future study.

Different age groupings (Table 2) show there was response transition in those in their late 40s, leading to a plateau in the antibody response to the rPvCSP-c. The level of antibody response seen by the mean responses increased significantly with age as expected because of people repeatedly exposed to the malaria parasite [44]. These results might also demonstrate that even low level and unstable transmission leads to a potent boosting of the antibody level and high-level antibody only at mature adulthood in residents of this region. Antibody levels were possibly indicative of previous exposures and the acquisition of parasitaemia limiting blood stage infection [45,46]. Previously, the vivax-infected patient serum levels did not differ significantly between the age groups which were different from present findings [47]. This might be because different serum samples used for response detection. Furthermore, this reactivity to native protein using both VK210 and VK247 sporozoites in an immunofluorescence assay with anti-rPvCSP-c antibodies were confirmed (Figure 5). These results suggested that rPvCSP-c was able to be serological marker, reacting with the majority of vivax-infected patient serum samples.

Since repeat region was involved B and T cells epitopes that different number of *csp* repeat of *P. vivax* patient serum against the chimeric protein was analysed. Although only limited known number of repeat region samples was shown, a negative correlation tendency between repeat number and immune responsiveness, which supported us a novel prospective way to design rPvCSP-c. In Figure 4B, all higher parasitaemia samples poorly recognized rPvCSP-c, the negative correlation between parasitaemia and rPvCSP-c, which may suggest the antibodies against rPvCSP-c in some degree inhibit overall parasite growth [48]. Previously, total IgG responses to PvCSP were positively with parasitaemia that was different from present findings [47], which might be also according to study population collected from different geographic areas. These observations surely required additional defined-cohort studies to address antibody levels-protection correlations.

Previous studies show low natural immune response against the native CSP by different geographic regions [49,50]. Comparatively, high immune response was analysed against synthetic peptides based on VK247 in different parts of Columbia [17]. Furthermore, natural immune responses in ROK to various types of PvCSP study showed that the sensitivity of them was highly detectable in enzyme-linked immunosorbent assays (ELISA) [51]. These results indicated rPvCSP-c was highly antigenic and was recognized by nature immune response antibodies confirms previous findings that sensitivity was 65% [28] (Table 1). Notably, the rPvCSP-c sensitivity of present test was higher than that tested using Brazilian serum samples, it might be because rPvCSP-c backbone was VK210 that was specific for VK210 allele in Korea. Nevertheless, these results suggested that rPvCSP-c proteins expressed by cell-free expression system and evaluated by protein array were stable. Hence, it shows the consistency of antibody level to rPvCSP-c from natural exposure although their construct is different. Together, these findings also confirm protein array platform is easy to handle and stable for detection immune responses instead of ELISA.

In conclusion, a soluble recombinant chimeric PvCSP protein has been expressed and purified successfully that includes conserved regions and variant regions of major two types (VK210 and VK247) of the *P. vivax* CSP. The specific serological reaction of rPvCSP-c to both VK210 and VK247 type vivax patient samples supported this chimeric protein potential as global serologic marker.

## Competing interests

The authors declare that they have no competing interests.

## Authors’ contributions

YC contributed to write the manuscript, to design and to conduct the experiments. JS produced sporozoite parasites and DI performed IFA. DHK, KSH, CSL and BW support technical advice for this study. TT constructed PvCSP-c plasmid DNA and expressed recombinant protein. ETH and TT conceived this study and contributed to write the manuscript and to review manuscript critically. All authors read and approved the final manuscript.
